# Castleman's disease in the head of the pancreas: report of a rare clinical entity and current perspective on diagnosis, treatment, and outcome

**DOI:** 10.1186/1477-7819-5-133

**Published:** 2007-11-20

**Authors:** Hongbei Wang, Rosemary L Wieczorek, Michael E Zenilman, Fidelina Desoto-Lapaix, Bimal C Ghosh, Wilbur B Bowne

**Affiliations:** 1 Department of Pathology, The State University of New York, Health Science Center of Brooklyn, NY, USA; 2 Department of Surgery, Department of Veterans Affairs, New York Harbor Health Care System, Brooklyn, NY, USA; 3 Department of Surgery, The State University of New York, Health Science Center of Brooklyn, Brooklyn NY, USA; 4 Department of Pathology, Department of Veterans Affairs, New York Harbor Health Care System, Brooklyn, NY, USA

## Abstract

**Background:**

Castleman's disease of the pancreas is a very rare condition that may resemble more common disease entities as well as pancreatic cancer.

**Case presentation:**

Here we report the case of a 58-year-old African American male with an incidentally discovered lesion in the head of the pancreas. The specimen from his pancreaticoduodectomy contained a protuberant, encapsulated mass, exhibiting microscopic features most consistent with localized/unicentric Castleman's disease. These included florid follicular hyperplasia with mantle/marginal zone hyperplasia along with focal progressive transformation of germinal centers admixed with involuted germinal centers.

**Conclusion:**

To date, eight cases of Castleman's disease associated with the pancreas have been described in the world literature. We report the first case of unicentric disease situated within the head of the pancreas. In addition, we discuss the diagnostic dilemma Castleman's disease may present to the pancreatic surgeon and review current data on pathogenesis, treatment, and outcome.

## Background

Localized lymphoid hyperplasia of the pancreas is rarely reported and is often indistinguishable from pancreatic neoplasms both clinically or radiographically [1,2]. Castleman's disease (CD), a morphologically distinct form of lymph node hyperplasia is very rare, and even more infrequent in the pancreas [3]. Currently, there are only eight reported cases in the literature [3-10]. In our case, the lesion within the head of the pancreas was presumed to be a pancreatic carcinoma, and a classical pancreaticoduodenectomy (PD) was performed. Pathological examination instead revealed changes consistent with a localized or unicentric hyaline-vascular (HV) variant of CD within the head of the pancreas. In this communication, we review the diagnosis, pathogenesis, treatment, and outcome for this rare clinical entity.

## Case presentation

**Figure 1:**
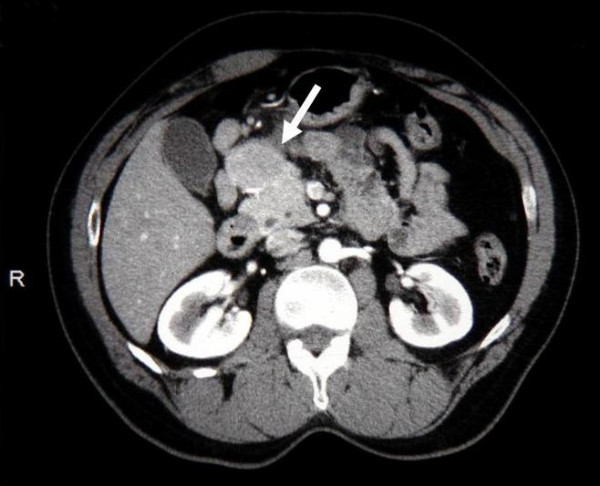
Computed tomography demonstrating enhancing mass protruding from the head of the pancreas. White arrow points to Castleman's disease in pancreas.

A 58-year-old African American male was seen in hepatology clinic for a surveillance liver ultrasound following a diagnosis of hepatitis C. The patient had experienced mild intermittent upper abdominal pain consistent with a history of gastroesophageal reflux disease. He had no weight loss, change in appetite or bowel habits, jaundice, or general malaise. Past medical history included hyperlipidemia, peptic ulcer disease, and hepatitis C (antibody positive, PCR negative). The patient's physical examination and laboratory studies were otherwise normal. A surveillance abdominal ultrasound demonstrated a well circumscribed mass measuring 3.4 × 2.9 cm within the head of the pancreas. A computed tomography (CT scan) further confirmed an enhancing mass involving the head of the pancreas (Figure 1). No evidence of extra-pancreatic disease was found on further evaluation. Although tumor-associated antigen levels (CEA, CA 19-9, and CA-125) were normal our differential diagnosis still included adenocarcinoma of the pancreas. Endoscopic ultrasound (EUS) guided needle biopsy was then performed but the pathological findings were non-diagnostic. Our concern for possible undetected malignancy remained and a diagnostic laparoscopy was performed followed by PD. The patient recovered well following the procedure and was discharged home on the 15^th ^postoperative day. He continues to receive close outpatient surveillance without additional treatment.

### Pathologic findings

**Figure 2:**
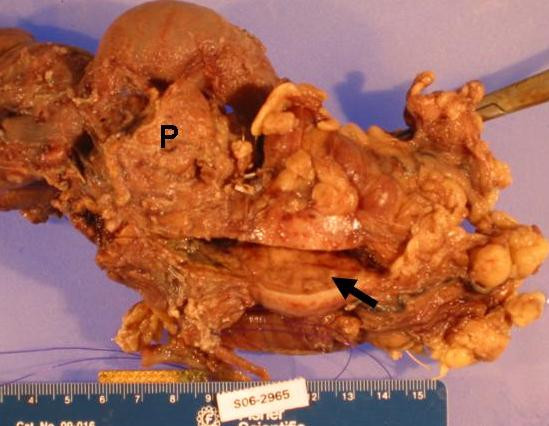
A protuberant 4 × 3 × 2.8 cm, partially encapsulated, oval mass within the superior border of the pancreatic head (P). Arrows points to tan-gray fleshy firm cut surface of mass.

**Figure 3:**
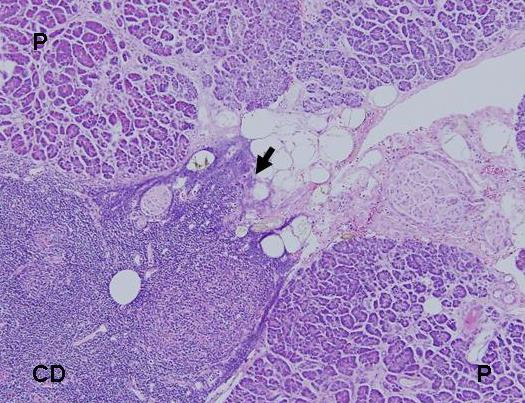
Castleman's disease (CD) infiltrating normal pancreatic tissue (P) (hematoxylin-eosin, original magnification × 100). Black arrow demonstrates infiltration of CD into normal pancreas.

Within the PD specimen, a protuberant 4 × 3 × 3 cm circumferential mass at the superior border of the pancreatic head was appreciated (Figure 2). The external surface of the mass was tan/gray, firm, and smooth. Cut sections of the mass also revealed a partially encapsulated large nodule with tan/gray fleshy to firm parenchyma infiltrating the pancreas at its distal portion. Surgical margins were clear (Figure 3). Regional lymph nodes were identified near the mass; microscopic examination revealed that all were hyperplastic lymph nodes. The remaining surgical margins were negative.

**Figure 4:**
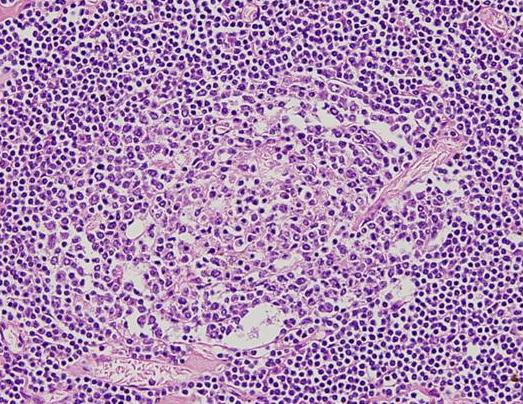
Hyaline-vascular-CD. Involuting germinal center surrounded by concentric rings of small lymphocytes penetrated by hyalinized vessel (hematoxylin-eosin, original magnification × 400).

This lymphoid lesion within the pancreatic head showed marked vascular proliferation and hyalinization of germinal centers. Involuted germinal centers were surrounded by concentric rings of small lymphocytes. i.e. "onion-skinning" penetrated by hyalinized vessels (Figure 4). Moreover, the interfollicular zones were rich in plasma cells and hyalinized vasculature. These features are consistent with localized hyaline-vascular type CD. Distribution of T and B cell markers (CD20, CD3, CD45) was normal and markers indicative of lymphoid malignancies (i.e. CD30, CD15, CD5, CD10, BCL-2) did not show evidence of such entities. Furthermore, PCR analysis of paraffin-embedded tissue revealed no clonal rearrangement of the immunoglobulin heavy chain or T-cell receptor gamma and beta chain genes. *MALT1 *(18q21) clonal rearrangement by FISH was negative. Together, these morphologic and immunohistochemical findings are most consistent with a diagnosis of CD.

## Discussion

Historically, CD, also known as angiofollicular lymph node hyperplasia, remains a rare and poorly understood disease characterized by massive growth of lymphoid tissue. CD was first described as a pathological entity in 1954 and later defined by Castleman *et al*., in 1956 [11]. A variety of terms have been used to describe this disorder, including giant lymph node hyperplasia, lymph node hamartoma, follicular lymophoreticuloma, benign giant lymphoma, angiomatous lymphoid hamartoma, and angiofollicular mediastinal lymph node hyperplasia. Flendrig and Schillings [12] described two basic histopathological subtypes and one mixed variant which Keller *et al*. [13] later designated HV, plasma cell (PC), and hyaline-vascular plasma cell "mixed" types (HV-PC). Categorization of CD into clinically relevant subtypes was proposed by McCarty *et al*. [14] and Gaba *et al*. [15] into unicentric and multicentric variants, respectively. In general, HV-CD is commonly associated with a localized -asymptomatic mass (76–91%) [15,16], while PC-CD is usually multicentric and symptomatic in 50% of patients.

Unicentric and multicentric CD differ in their clinical presentation and distribution of adenopathy. In a series from Memorial Sloan-Kettering Cancer Center (MSKCC) [16], patients with unicentric CD were frequently discovered incidentally with symptoms mostly arising from mass compression. Masses found in unicentric disease were usually centrally located in the abdomen and pelvis (54%) and mediastinum (31%), whereby systemic symptoms, central and peripheral adenopathy, organomegaly, and associated abnormal laboratory values (elevated erythrocyte sedimentation rates [ESR], interleukin-6 [IL-6], anemia and polyclonal gammaglobulinemia) were predominant in the multicentric variant.

Our current understanding of the pathogenesis of CD points to reactive follicular hyperplasia in response to an unknown antigenic stimulus [17]. In the current report, the role of HCV infection (positive serology for anti-HCV) in the pathogenesis of HV-CD is possible. This view has been shared by others. Multicentric PC-CD has been previously reported to be associated with concurrent HCV infection in a child with Klinefelter's syndrome and nonregulated antibody production mimicking systemic lupus erythematosus stabilized by interferon-alpha therapy suggesting an association with HCV [18]. Likewise, a report associating reactive lymphoid hyperplasia representing a pseudo-lymphoma of the liver was detailed in a patient with chronic hepatitis C [19]. Moreover, the potential role of HCV infection in an antigen-driven lymphoproliferative model for the pathogenesis of unrelated lymphoproliferative disease entities including non-Hodgkin's lymphoma, marginal zone B-cell lymphoma, or extranodal marginal zone B-cell lymphoma of mucosa-associated lymphoid tissue (MALT) further supports this hypothesis. Nevertheless, the association of concurrent HCV infection and HV-CD requires further investigation. In addition, other etiologies including chronic low grade inflammation [11], harmartomatous process [20,21], immunodeficiency [22-24], and autoimmunity [25] have all been proposed as possible pathogenic mechanisms. Epstein-Barr virus, Toxoplasma, and *Mycobacterium tuberculosis *also have been linked to this disorder [13,25]. Interestingly, lymph nodes from various animal models and patients with CD implicate IL-6 as a causative agent for the commonly observed systemic manifestations [20,26-29].

The initial challenge in CD remains in establishing the diagnosis. Considerable imprecision exists in distinguishing CD from other lymphoid and non-lymphoproliferative disorders that it may resemble clinically and pathologically [30]. Therefore, in the appropriate clinical setting, the diagnosis of CD must be considered, after investigating and excluding more common causes of lymphadenopathy or associated neoplastic processes. Intra-operatively, distinguishing CD involving the head of the pancreas from pancreatic carcinoma may be problematic and strong suspicion of pancreatic malignancy as in our case may prompt traditional surgical removal. Possible clues for the pancreatic surgeon may include evidence of an encapsulated pancreatic mass that is well-circumscribed, and smooth; an uncommon finding for pancreatic adenocarcinoma. Indeed, the diagnosis ultimately rests on precise pathologic investigation.

Unicentric CD is largely the HV- type. HV-CD typically occurs as an isolated lymph node mass or regional adenopathy. These patients are frequently asymptomatic. From a histopathologic standpoint, "lollipop" follicles; germinal centers surrounded by circumferentially arranged layers ("onion skin") of small lymphocytes interconnected by a prominent vascular stroma are characteristic pathological findings. In this report, our patient had all the classical features of unicentric HV-CD. Conversely, the PC-CD subtype demonstrates continuous sheets of dense plasma cells and a less vascular interfollicular stroma surrounding the germinal centers [13].

Unicentric CD specifically involving the pancreas is extremely rare, with only eight cases described worldwide [3-10] (Table 1). The majority of these cases were of the HV-subtype and discovered incidentally. The PC-subtype typically presents with systemic symptoms. In those cases with patient follow-up it appears that resection may offer short-term control of disease with resolution of associated symptomatology.

**Table 1:** Summary of patients with unicentric Castleman's disease involving the pancreas

**Author**	**Site**	**Symptoms**	**Subtype**	**Treatment**	**Outcome**
Goetze^3^	Tail of Pancreas	No	HV-CD	DP	NED (2 Yrs)
Erkan^4^	Peripancreatic	Abdominal pain*	PC-CD	Enucleation	NED (1 yr)
Baikovas^5^	Peripancreatic	No	HV-CD	Excision	NA
Lepke^6^	Body and Tail	No	HV-CD	STP	NA
Corbisier^7^	Peripancreatic	Abdominal pain	HV-CD	Excision	NA
LeVan^8^	Tail of Pancreas	Back pain	HV-CD	DP	NA
LeBorgne^9^	Uncinate Process	Systemic	PC-CD	PD	NED+ (11 mos)
Brossard^10^	Tail of Pancreas	Systemic	PC-CD	DP	+
Current Report	Head of Pancreas	No	HV-CD	PD	NED (1 yr)

HV-CD: hyaline vascular Castleman's disease; PC-CD: plasma cell Castleman's disease;

* Elevated erythrocyte sedimentation rate, C-reactive protein, and hypergammaglobulinemia; DP: distal pancreatectomy; STP; subtotal pancreatectomy;

PD: pancreaticoduodenectomy; + resolution of systemic symptoms;

NED: No evidence of disease; NA: Not available

Additional studies have further clarified the natural history following resection. From a consensus standpoint, surgical resection is the mainstay of treatment for unicentric CD with most reports describing complete resection as being curative. In a recent report from MSKCC [16], complete resection of unicentric disease was curative for all patients regardless of histologic subtype. Likewise, Keller *et al*. [13] retrospectively examined 61 patients with unicentric disease who were treated with surgery over a 20 year period. Their study demonstrated that for patients with unicentric HV-CD, complete resection offered the best chance for cure. In certain cases, if complete resection is not possible, partial resection or observation with long term follow-up may be useful. As reported, radiographic examination (CT or MRI) along with arteriography and embolization has been used to facilitate surgical excision and minimize intraopertative bleeding, which can be profuse [31-34].

A limited number of reports address the response of CD to radiotherapy (2700–4500 cGy) administered to involved sites which has resulted in remission of disease in isolated cases [28,35-42], but failed in others [13,15,43]. Keller *et al*. [13], reported on four patients with unicentric-HV disease who were treated with 1800–4300 cGy with no response. Notably, a complete response of the unicentric-HV variant was reported by Sethi *et al*. [40], in a patient with systemic symptoms treated with 4000 cGy. However, irradiation was noted to produce a range of favorable responses in all patients of PC or mixed histology [35,36,38,39,42]. These results point to a better response when radiotherapy is administered at an earlier, more active stage of disease (PC and mixed- HV-PC unicentric subtypes), rather than with the later, less metabolically active HV-subtype.

Subsequently, close follow-up and periodic surveillance are necessary to detect concurrent or ensuing malignant lesions (lymphoproliferative disease and vascular neoplasms) [44,45] associated with CD. Notably, local recurrence has been reported as long as 11 years after complete resection.

In contrast, no effective therapy has been established for multicentric disease which is widely viewed as a systemic disease. Among very rare cases, surgery may play a limited role for cases of palliation of systemic symptoms. Steroids, single-agents, or combination chemotherapy, plus immunotherapy are currently being employed with results ranging from rare cases of complete remission, sustained manifestations of disease, to aggressive disease biology with rapidly fatal outcomes [43,46,47]. The worst prognosis is for patients with multicentric disease, PC-subtype, and clinical signs of neuropathy. This group appears refractory to all therapy [46]. Long term survival may be possible in multicentric patients harboring the HV- subtype.

In this report, our patient underwent a PD for a presumed pancreatic cancer. In most cases, surgeons who treat pancreatic cancer will usually proceed to surgery without biopsy if the evidence of malignancy is strong. In this case, the initial biopsy did not demonstrate cancer. However, a study that is negative for tumor should not always be interpreted as meaning that no tumor exists. In this instance, a curable form of CD was resected. Due to the rarity of this disease, a diagnosis of CD will usually occur well after pathologic exclusion of other more common disease entities.

## Conclusion

CD is a poorly understood disease that creates both a diagnostic and therapeutic dilemma for surgeons. Complete surgical resection of unicentric disease at the time of presentation is likely to afford the best chance for cure. Radiation therapy has been used with varied success in patients who are poor surgical candidates or in those with unresectable lesions. Long term follow-up is necessary with regard to malignant sequelae. The role of surgery in multicentric disease is limited and should not be considered a realistic treatment option. Systemic therapy in the form of steroids, single or multiple drug chemotherapies have all been used with varied success. However, there is no evidence for one approach being more consistently effective. A better understanding of the pathogenesis, natural history, and ultimately diagnosis of this disorder may lead to improvement over the current modalities available for treatment.

## Competing interests

The author(s) declare that they have no competing interests.

## Authors' contributions

**HW**-Drafting manuscript, acquisition of data, **RLW**-acquisition of data, analysis and interpretation, **MEZ**-revising of manuscript, **FDL**-acquisition of data, analysis and interpretation, **BCG**-revising of manuscript, **WBB**-drafting of manuscript, analysis and interpretation, revising manuscript.

All authors read and approved the final manuscript.
